# A new diagnostic tool for brain disorders: extracellular vesicles derived from neuron, astrocyte, and oligodendrocyte

**DOI:** 10.3389/fnmol.2023.1194210

**Published:** 2023-08-09

**Authors:** Xueying Wang, Huihui Yang, Chunyu Liu, Kefu Liu

**Affiliations:** ^1^Center for Medical Genetics and Hunan Key Laboratory of Medical Genetics, School of Life Sciences, Central South University, Changsha, Hunan, China; ^2^Department of Psychiatry, State University of New York Upstate Medical University, Syracuse, NY, United States

**Keywords:** neurodegenerative disorders, psychiatric disorders, NDEs, ADEs, ODEs, diagnosis, biomarker

## Abstract

Brain disorders are the leading cause of disability worldwide, affecting people’s quality of life and causing economic burdens. The current clinical diagnosis of brain disorders relies solely on individual phenotypes and lacks accurate molecular biomarkers. An emerging field of research centers around extracellular vesicles (EVs), nanoscale membrane vesicles which can easily cross the blood–brain barrier. EVs in the blood are derived from various tissues, including the brain. Therefore, purifying central nervous system (CNS)-derived EVs from the blood and analyzing their contents may be a relatively non-invasive way to analyze brain molecular alterations and identify biomarkers in brain disorders. Recently, methods for capturing neuron-derived EVs (NDEs), astrocyte-derived EVs (ADEs), and oligodendrocyte-derived EVs (ODEs) in peripheral blood were reported. In this article, we provide an overview of the research history of EVs in the blood, specifically focusing on biomarker findings in six major brain disorders (Alzheimer’s disease, Parkinson’s disease, schizophrenia, bipolar disorder, depression, and autism spectrum disorder). Additionally, we discuss the methodology employed for testing CNS-derived EVs. Among brain disorders, Alzheimer’s disease has received the most extensive attention in EV research to date. Most studies focus on specific molecules, candidate proteins, or miRNAs. Notably, the most studied molecules implicated in the pathology of these diseases, such as Aβ, tau, and α-synuclein, exhibit good reproducibility. These findings suggest that CNS-derived EVs can serve as valuable tools for observing brain molecular changes minimally invasively. However, further analysis is necessary to understand the cargo composition of these EVs and improve isolation methods. Therefore, research efforts should prioritize the analysis of CNS-derived EVs’ origin and genome-wide biomarker discovery studies.

## 1. Introduction

Human brain disorders, including neurodegenerative and psychiatric disorders (Naz and Siddique, 2020), are the leading cause of disability worldwide, imposing a severe burden on families and society. However, the current diagnostic criteria for these disorders are all based on evaluating clinical symptoms, making it crucial to find objective biological indicators. Extensive research on the pathological mechanisms of neurodegenerative disorders has mainly focused on amyloidosis, pathological accumulation of tau protein, and aggregation of α-synuclein (Dugger and Dickson, 2017). Presently, neuropathological assessment during autopsy remains the sole definitive method for identifying disease types. Neuroimaging studies have revealed structural abnormalities in the brains of individuals with psychiatric disorders (Opel et al., 2020). Establishing a less invasive diagnostic method capable of capturing pathological brain changes would greatly aid in clinical molecular classification and drug discovery.

Extracellular vesicles (EVs) are nanoscale membrane vesicles secreted by cells and widely distributed in various biological fluids. These EVs can be categorized into several subtypes: (1) exosomes (~50–150 nm in diameter), (2) retroviruses (80–150 nm), (3) microvesicles, ectosomes, or microparticles (~100–1,000 nm), and (4) apoptotic EVs and apoptotic bodies (~100–5,000 nm) (Mathieu et al., 2019). EVs harbor a variety of biomolecules, including protein, miRNA, mRNA, and more, which can be transported between different cells, tissues, or organs and participate in various physiological processes. In recent years, studies have found that EVs’ abnormal secretion and cargos are closely related to the pathogenesis of central nervous system (CNS) diseases. What’s more, because of their intercellular communication abilities and the capacity to cross the blood–brain barrier (BBB) (Banks et al., 2020; Morales-Prieto et al., 2022), EVs have emerged as potential tools for breakthroughs in the clinical diagnosis of brain disorders. Recent studies have shown that EVs may play a role in the abnormal accumulation or degeneration of proteins in the brains of patients with neurodegenerative disorders through secretion and transmission between cells (Yuyama et al., 2008; Emmanouilidou et al., 2010; Saman et al., 2012; Nonaka et al., 2013). In the case of psychiatric disorders, transplantation of serum-derived EVs from schizophrenia (SCZ) patients into mice resulted in SCZ-like behavioral and molecular phenotypes (Du et al., 2021). Moreover, peripheral injection of blood-derived EVs from healthy controls (HC) into chronic unpredictable mild stress (CUMS) mice alleviated depression-like behavior (Wei et al., 2020).

Analyzing changes in biomolecules within CNS-derived EVs isolated from peripheral tissues may capture alterations occurring in the brain. The utilization of EVs has the potential to facilitate minimally invasive liquid biopsies for clinical diagnosis and tracking of disease progression. However, due to the heterogeneity of EVs and their diverse origins, measuring molecular changes in the brain using total EVs in the bloodstream poses challenges. Purifying specific EVs derived from the brain may circumvent these limitations and enable the investigation of their cargos as biomarkers of brain disorders. Recent studies have reported the existence of neuron-derived EVs (NDEs), astrocyte-derived EVs (ADEs), and oligodendrocyte-derived EVs (ODEs) in the brain (Zou et al., 2022).

In this review, we focus on these three sources of EVs and compile a comprehensive summary of the historical background and biomarker studies conducted in major neurodegenerative [Alzheimer’s disease (AD) and Parkinson’s disease (PD)] and psychiatric disorders [SCZ, bipolar disorder (BD), depression, and autism spectrum disorder (ASD)]. Furthermore, we discuss the limitations inherent in existing studies and propose future directions for research.

## 2. The research history of blood EVs derived from the central nervous system in brain disorders

Harding and Stahl (1983) and Pan and Johnstone (1983) made the initial discovery of small vesicles (~50 nm) secreted from reticulocytes, which they named “exosomes.” These exosomes were found to play an important role in the reticulocyte transferrin receptor cycle process. Later, Zitvogel et al. (1998) and Théry et al. (2001) found that dendritic cells could secrete exosomes and activate the T cell immune response. Fevrier et al. (2004) discovered the propagation of the first pathological protein, prion protein (PrP), through exosomes. These findings implied the potential relevance of exosomes in both normal and pathological brain physiology. It also raised the possibility of using exosomes to explore disease pathology and develop diagnostic tools for clinical applications. In accordance with the Minimal information for studies of extracellular vesicles 2018 (MISEV2018) recommendations and considering the imprecise methods for isolating pure exosomes, we will use the term “EVs” to replace “exosomes” in the subsequent description.

Fauré et al. (2006) found that neurons and astrocytes in the rat cortical primary cultures could secrete EVs. Through the LC–MS/MS and western blot analyses, they identified specific proteins present in EVs secreted by astrocytes (highly expressing glutamine aspartate transporter, GLAST) and neurons (highly expressing L1 cell adhesion molecule, L1CAM) in the brain. In 2007, it was confirmed that oligodendrocytes also secrete EVs, which carry abundant proteolipid protein (PLP) and 2’3’-cyclic-nucleotide phosphodiesterase (CNP). These findings laid the groundwork for subsequent studies on NDEs, ADEs, and ODEs (Krämer-Albers et al., 2007).

CNS-derived EVs have been studied in clinical blood samples since 2014. Shi et al. (2014) and Fiandaca et al. (2015) independently established an immunoaffinity capturing protocol to isolate L1CAM-containing EVs (referred to as NDEs) from plasma. L1CAM is a cell adhesion molecule highly expressed in neurons. Two years later, Goetzl et al. (2016b) made progress in ADEs using mouse anti-human GLAST biotinylated antibodies. The study examined the association between cargo proteins of plasma ADEs and AD pathology. The first clinical study on ODEs was published in 2019 (Ohmichi et al., 2019). Since then, more and more researchers have carried out clinical research on these special EVs, as described in the following sections.

## 3. Research on NDEs, ADEs, and ODEs relevant to clinical diagnoses of brain disorders

**Figure 1:**
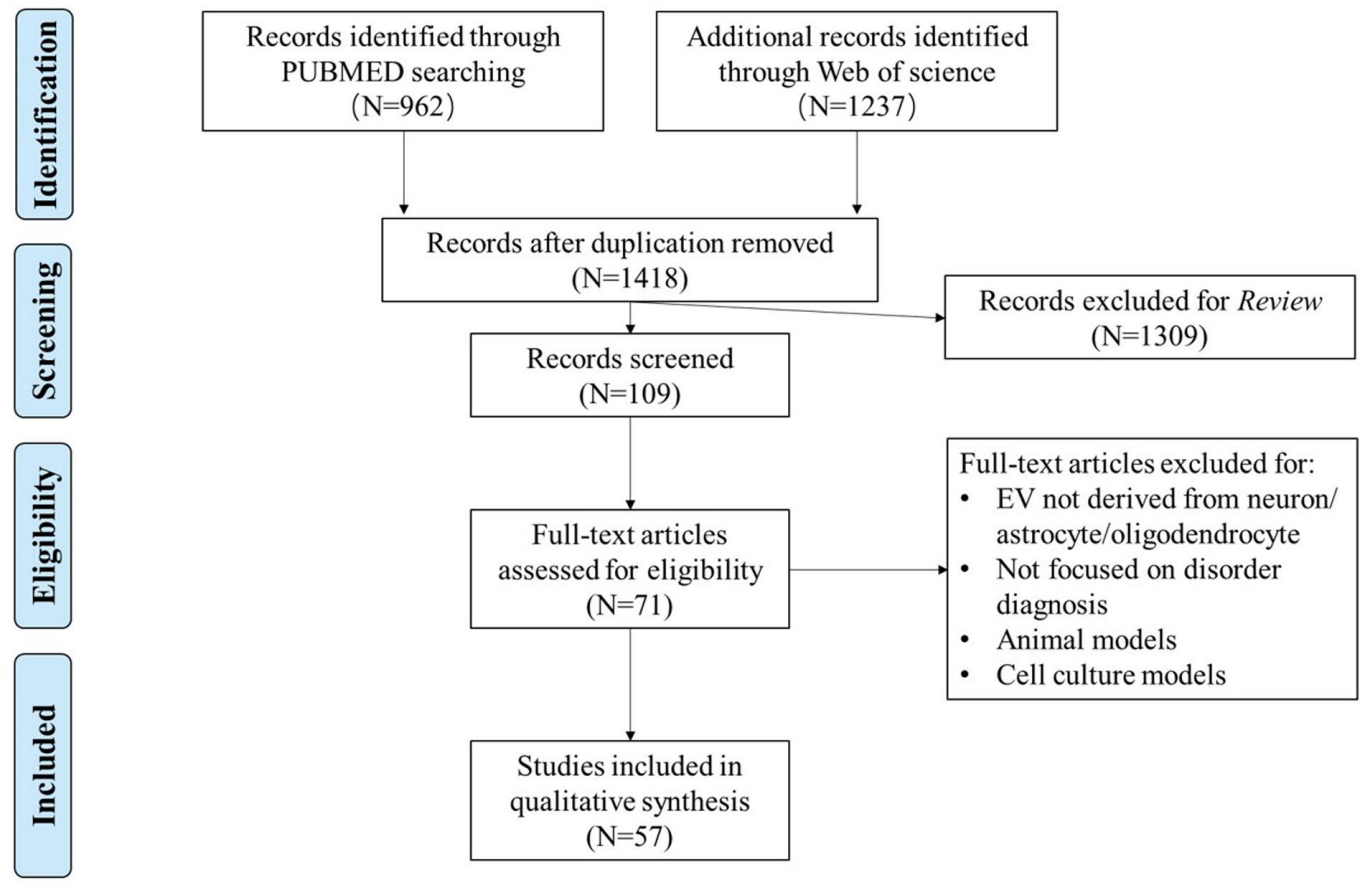
Flowchart of data selection using the Preferred Reporting Items for Systematic Reviews and Meta-Analyses (PRISMA).

To evaluate the potential role of molecules from NDEs, ADEs, and ODEs in clinical diagnosis, we conducted a systematic review of published studies that detected cargos in these specific EVs in the blood of patients with brain disorders. PubMed and Web of Science were searched with the following search builder: *((extracellular vesicle) OR (extracellular vesicles) OR (exosome) OR (exosomes) OR (exosomal) OR (exomeres) OR (microparticles) OR (apoptotic bodies) OR (retroviruses)) AND ((neurodegenerative disease) OR (neuropsychiatric disorder) OR (mental disorder) OR (psychiatry disorder) OR (psychotic disorder) OR (psychiatric disorder) OR (neurodevelopmental disorder) OR (neurological disease) OR (neurological disorder) OR (psychosis) OR (#DISORDER#))*. #DISORDER# is one of the six major brain disorders: AD, PD, SCZ, BD, depression, and ASD. The inclusion criteria were the following: (1) published in English before April 2022; (2) assessed EVs derived from neuron/astrocyte/oligodendrocyte; and (3) focused on clinical diagnosis. Studies on animal and cell culture models were excluded. The literature selection followed the standard of Preferred Reporting Items for Systematic Reviews and Meta-Analyses (PRISMA) (Liberati et al., 2009; Figure 1).

A total of 57 publications that met our inclusion and exclusion criteria were included in our review (Supplementary Table S1). These studies covered AD (number of studies, *n* = 30), PD (*n* = 16), SCZ (*n* = 4), BD (*n* = 1), depression (*n* = 5), and cross-disorder studies (AD and PD, *n* = 1). Notably, there are no NDEs/ADEs/ODEs-related studies on ASD or other brain disorders. Among the included studies, 54 of the 57 papers examined NDEs, while only 10 and 3 studies have investigated ADEs and ODEs, respectively. Regarding the types of molecules analyzed in the included literature, only five papers reported changes in miRNAs within EVs, while the rest explored protein molecules. Two studies did not analyze the cargos of EVs. To evaluate the reproducibility of the reported changes in molecules derived from these specific EVs in the cases compared to controls, we examined the consistency of the reported changes in each disorder by the presence of significant results (*p* < 0.05) consistent in more than one independent study. We summarized those molecules explored in two or more studies in Table 1, including the related study subjects, sample sizes, and the direction of change. It should be emphasized that, based on the currently included studies, it is not possible to assess the reproducibility of the factors explored in the ADEs/ODEs related studies, as none of these factors were explored in more than two related studies. A comprehensive description of the results from the included literature can be found in Supplementary Table S1.

**Table 1:** SUMMARY of factors explored by two or more studies.

Factors	Type of EVs	Study objects	Direction of change	PubMed ID (Sample size)
Alzheimer’s disease				
Aβ42	NDEs	AD vs. HC	Increase	25130657 (57 vs. 57), 27231710 (20 vs. 10), 27408937 (10 vs. 10), 27511944 (12 vs. 10), 31422798 (28 vs. 29), 32741361 (88 vs. 80), 32790155 (31 vs. 15), 35287177 (36 vs. 41)
		AD vs. pre-clinical AD	Increase	27408937 (10 vs. 20), 31422798 (28 vs. 25), 32741361 (88 vs. 87), 35287177 (36 vs. 97)
		Pre-clinical AD vs. HC	Increase	27408937 (10 vs. 20), 30372675 (31 vs. 36; 40 vs. 30), 31422798 (25 vs. 29), 32741361 (87 vs. 80), 35287177 (29 vs. 41)
		Longitudinal sets (as the disease progresses)	Increase	25130657 (24), 27231710 (20)
			No change	31305918 (128)
Total tau	NDEs	AD vs. HC	Increase	25130657 (57 vs. 57), 31422798 (28 vs. 29), 32679907 (18 vs. 23), 35287177 (36 vs. 41)
		AD vs. pre-clinical AD	Increase	31422798 (28 vs. 25), 32679907 (18 vs. 29), 35287177 (36 vs. 97)
		Pre-clinical AD vs. HC	Increase	31422798 (25 vs. 29), 35287177 (29 vs. 41)
		Longitudinal sets (as the disease progresses)	Increase	25130657 (24)
			No change	31305918 (128)
p-T181-tau	NDEs	AD vs. HC	Increase	25130657 (57 vs. 57), 27231710 (20 vs. 10), 27408937 (10 vs. 10), 27511944 (12 vs. 10), 31422798 (28 vs. 29), 32790155 (31 vs. 15), 35287177 (36 vs. 41)
			No change	29495441 (20 vs. 10)
		AD vs. pre-clinical AD	Increase	31422798 (28 vs. 25), 35287177 (36 vs. 97)
			No change	27408937 (10 vs. 20), 29495441 (20 vs. 10)
		Pre-clinical AD vs. HC	Increase	27408937 (20 vs. 10), 31422798 (25 vs. 29), 35287177 (29 vs. 41)
			No change	29495441 (10 vs. 10)
		Longitudinal sets (as the disease progresses)	Increase	25130657 (24), 27231710 (20), 31305918 (128)
p-S396-tau	NDEs	AD vs. HC	Increase	25130657 (57 vs. 57), 27231710 (20 vs. 10), 27408937 (10 vs. 10), 27511944 (12 vs. 10)
			No change	32790155 (31 vs. 15)
		Pre-clinical AD vs. HC	Increase	27408937 (20 vs. 10), 27511944 (12 vs. 10)
			No change	30372675 (31 vs. 36; 40 vs. 30)
		Longitudinal sets (as the disease progresses)	Increase	25130657 (24)
			No change	27231710 (20)
NRGN	NDEs	AD vs. HC	Decrease	27231710 (20 vs. 10), 27408937 (10 vs. 10), 27601437 (12 vs. 28), 32776690 (28 vs. 29)
		AD vs. pre-clinical AD	Decrease	27408937 (10 vs. 20), 32776690 (28 vs. 25)
		Pre-clinical AD vs. HC	Decrease	27408937 (20 vs. 10), 30372675 (31 vs. 36; 40 vs. 30), 32776690 (25 vs. 29)
		Longitudinal sets (as the disease progresses)	Decrease	27231710 (20), 27601437 (9), 32776690 (160)
Synaptotagmin	NDEs	AD vs. HC	Decrease	27601437 (12 vs. 28), 32776690 (28 vs. 29)
		Pre-clinical AD vs. HC	Decrease	30372675 (31 vs. 36; 40 vs. 30), 32776690 (25 vs. 29)
		Longitudinal sets (as the disease progresses)	Decrease	27601437 (9), 32776690 (160)
GAP43	NDEs	AD vs. HC	Decrease	27601437 (12 vs. 28), 32776690 (28 vs. 29)
		Pre-clinical AD vs. HC	Decrease	32776690 (25 vs. 29)
			No change	30372675 (31 vs. 36; 40 vs. 30)
		Longitudinal sets (as the disease progresses)	Decrease	27601437 (9), 32776690 (160)
SNAP25	NDEs	AD vs. HC	Decrease	30680692 (24 vs. 17), 32776690 (28 vs. 29), 35287177 (36 vs. 41)
		AD vs. pre-clinical AD	Decrease	32776690 (28 vs. 25), 35287177 (36 vs. 97)
		Pre-clinical AD vs. HC	Decrease	32776690 (25 vs. 29), 35287177 (29 vs. 41)
Cathepsin D	NDEs	AD vs. HC	Increase	26062630 (26 vs. 26), 27231710 (20 vs. 10)
		Longitudinal sets (as the disease progresses)	Increase	26062630 (20), 27231710 (20)
REST	NDEs	AD vs. HC	Decrease	26273689 (24 vs. 24), 27231710 (20 vs. 10), 27408937 (10 vs. 10)
		Longitudinal sets (as the disease progresses)	Decrease	26273689 (16)
Parkinson’s disease				
α-synuclein	NDEs	PD vs. HC	Increase	24997849 (267 vs. 215), 30692923 (39 vs. 40), 32150777 (53 vs. 21), 32236821 (93 vs. 85), 32273329 (275 vs. 144), 32945162 (20 vs. 20), 33217562 (32 vs. 40), 33826157 (290 vs. 191), 33991233 (50 vs. 51)

### 3.1. Neurodegenerative disorders

Neurodegenerative disorders are associated with several well-established molecular changes in the brain, including β-amyloid (Aβ), tau protein and inflammatory factors abnormalities, mitochondrial dysfunction, and neurotransmitter imbalance. Some of these molecules are frequently detected in CNS-derived EVs to evaluate their potential roles for disease diagnosis. To evaluate the reproducibility of these biomarker changes in CNS-derived EVs, we examined the consistency of these molecules across multiple studies.

#### 3.1.1. Alzheimer’s disease

AD is the most extensively studied brain disorder involving EVs to date. Currently, the pathology of AD is hypothesized and verified by the presence of Aβ plaques and hyperphosphorylated tau (p-tau) tangles (Serrano-Pozo et al., 2011). The International Working Group (IWG)-2 criteria and the National Institute on Aging-Alzheimer’s Association (NIA-AA) framework (Dubois et al., 2014; Jack et al., 2018) propose cerebrospinal fluid (CSF)-Aβ and CSF-tau as diagnostic biomarkers for AD. Consequently, these neuropathological alterations have been extensively studied in the NDEs’ research on AD.

Twenty-six studies were assessed (Supplementary Table S1), including 12 studies on Aβ42 and 14 on tau in the NDEs of AD. Most studies found that Aβ42 protein levels in NDEs continuously increased with the progression of AD [AD vs. pre-clinical AD/HC (Fiandaca et al., 2015; Abner et al., 2016; Goetzl et al., 2016b; Winston et al., 2016, 2018; Jia et al., 2019; Gu et al., 2020; Zhao et al., 2020; Chi et al., 2022), as well as pre-clinical AD to HC (Winston et al., 2016, 2018; Jia et al., 2019; Zhao et al., 2020; Chi et al., 2022)]. It is also evidenced in the longitudinal studies (Fiandaca et al., 2015; Abner et al., 2016), although one study reported no significant changes (Kapogiannis et al., 2019b). The samples of these longitudinal studies were obtained from preclinical AD to AD. Other Aβ-related molecules, such as Aβ40 (Zhao et al., 2020), soluble amyloid precursor protein sAPPα and sAPPβ (Goetzl et al., 2016b), have also been studied. Interestingly, one study (Jia et al., 2019) found a strong negative correlation between Aβ42 in NDEs and CSF. CSF Aβ42 is a recommended diagnostic marker for AD (Dubois et al., 2021). This implies that NDEs Aβ42 may reflect CSF Aβ42 levels. Above all, these results provide evidence and support the potential of NDEs Aβ42 as an AD biomarker.

Tau protein appears later than Aβ in the brains of AD patients and is also a typical feature in a series of diseases called tauopathies. Tau proteins exist in multiple forms, with total tau, p-T181-tau, and p-S396-tau being widely studied. Similar to Aβ42, most studies have reported significantly higher levels of total tau, p-T181-tau, and p-S396-tau in NDEs of AD compared to pre-clinical AD/HC (Fiandaca et al., 2015; Abner et al., 2016; Goetzl et al., 2016b; Winston et al., 2016; Jia et al., 2019; Gu et al., 2020; Nam et al., 2020; Chi et al., 2022) and pre-clinical AD compared to HC (Goetzl et al., 2016b; Winston et al., 2016; Jia et al., 2019; Chi et al., 2022). It has also been confirmed in longitudinal studies (Fiandaca et al., 2015; Abner et al., 2016; Kapogiannis et al., 2019b). However, some studies found no significant changes in total tau (Kapogiannis et al., 2019b) and p-S396-tau (Abner et al., 2016) expression levels in the NDEs in longitudinal cohorts. Four studies (Winston et al., 2016, 2018; Guix et al., 2018; Gu et al., 2020) reported that the levels of p-T181-tau and p-S396-tau showed no significant difference between AD and pre-clinical AD/HC or pre-clinical AD and HC. Despite this, other studies have shown that p-S202-tau and p-T231-tau proteins are also higher in different stages of dementia (Kapogiannis et al., 2019b; Nam et al., 2020).

Based on accumulating evidence, several proteins have been implicated in AD or even in its asymptomatic stage (Kvartsberg et al., 2015; Casaletto et al., 2017). Neurogranin (NRGN), synaptotagmin, growth-associated protein 43 (GAP43), synaptosome-associated protein 25 (SNAP25), repressor element 1-silencing transcription factor (REST), and cathepsin D have been extensively studied. Except for cathepsin D, these proteins showed a significant decline compared to normal controls, either in AD, in pre-clinical AD, or in longitudinal studies (Goetzl et al., 2015b, 2016a; Abner et al., 2016; Winston et al., 2016, 2018; Agliardi et al., 2019; Jia et al., 2021; Chi et al., 2022). On the other hand, Cathepsin D has been found to significantly increase in AD (Goetzl et al., 2015a; Abner et al., 2016). However, one study found inconsistent results, which was conducted by Winston et al. (2018), and it found no significant difference in GAP43 levels between pre-clinical AD and HC.

Other proteins and miRNAs reported to be associated with AD include synaptopodin, neuroligin 1 (NLGN1), lysosome-associated membrane protein 1 (LAMP-1), miR-132, and others. These molecules have shown significant changes in AD (Goetzl et al., 2015a, 2016a, 2018a; Winston et al., 2018; Cha et al., 2019). However, it is worth noting that these findings are based on individual studies, and the reproducibility of the results has yet to be assessed.

While most studies have focused on candidate proteins or miRNAs for AD, only three studies have utilized a high-throughput genome-wide approach to identify biomarkers in NDEs. Mass-spectrum-based proteomics did not detect any previously mentioned proteins but identified new proteins, including complement-related proteins and hemoglobin (Arioz et al., 2021; Zhong et al., 2021). Serpente et al. (2020) recruited 20 AD patients and 20 HC and performed high-throughput detection of miRNAs in their plasma NDEs. They found significant differences in the levels of miR-23a-3p, miR-223-3p, miR-100-3p, and miR-190-5p between AD patients and controls.

There are fewer studies on ADEs than on NDEs in AD. A variety of proteins have been detected in ADEs, including β-site amyloid precursor protein-cleaving enzyme 1 (BACE-1) (Goetzl et al., 2016b), complement effector and regulatory proteins (Goetzl et al., 2018b; Winston et al., 2019), and some neuroinflammation-related proteins (Goetzl et al., 2018b). Nevertheless, these protein molecules have only been reported in single studies.

No ODEs-related clinical studies have been performed on AD samples, but one study (Goetzl et al., 2019) isolated EVs derived from chondroitin sulfate proteoglycan 4 (CSPG4) type oligodendrocyte precursor cells. This study characterized some growth factors in AD with specific EVs. They found that the levels of hepatocyte growth factor (HGF), fibroblast growth factors (FGFs)-2 and −13, and type 1 insulin-like growth factor (IGF-1) were significantly lower in AD patients than in controls. However, there was no significant change between the levels before and after cognitive impairment in AD patients.

In summary, the research on AD has mainly focused on the proteins involved in AD pathologies, such as Aβ and tau. Studies have also investigated other proteins related to abnormal functions, such as synaptic dysfunction, glucose metabolism, autophagy-lysosomal system, neuroinflammatory dysfunctions, and so on. Most of the proteins tested were unique to each study, except for Aβ, tau, NRGN, synaptotagmin, GAP43, SNAP25, cathepsin D, and REST, which had consistent results across several studies (Table 1). NDEs have been studied much more intensively than other CNS-derived EVs.

#### 3.1.2. Parkinson’s disease

PD is the second most common neurodegenerative disorder worldwide. In PD studies, a significant focus has been placed on investigating changes in α-synuclein protein levels in CNS-derived EVs. A large deposit of α-synuclein, called Lewy body (LB), is one of the neuropathological hallmarks of PD (Shults, 2006; Braak and Del Tredici, 2017). Several studies consistently reported a significant increase in α-synuclein protein levels in EVs of PD patients compared to controls (Shi et al., 2014; Zhao et al., 2018; Fu et al., 2020; Jiang et al., 2020, 2021; Niu et al., 2020; Zou et al., 2020; Agliardi et al., 2021; Dutta et al., 2021; Table 1). Additionally, DJ-1 (Zhao et al., 2018), tau (Shi et al., 2016), and miR-155 (Arioz et al., 2021) were found to be increased in plasma NDEs of PD compared to controls. Insulin resistance-related proteins (Athauda et al., 2019; Chou et al., 2020) showed no significant changes between PD and controls. These molecules have been reported to be affected by α-synuclein (Burré et al., 2010) or act as regulators of α-synuclein, leading to the inflammatory response in PD (Thome et al., 2016).

Regarding the use of ODEs as a diagnostic tool for PD, three studies have recently been reported (Ohmichi et al., 2019; Yu et al., 2020; Dutta et al., 2021). Two of these studies explored the characteristics of α-synuclein in ODEs, but the results were inconsistent. Yu et al. (2020) found no change in α-synuclein levels between patients and controls, while Dutta et al. (2021) reported an increase in α-synuclein levels in PD.

In addition to candidate gene studies, two studies have employed a genome-wide approach in NDEs. Zou et al. (2020) found that the Linc-POU3F3 in plasma NDEs of PD patients was upregulated compared to normal controls through microarray analysis. Another study (Anastasi et al., 2021) characterized the proteomes of plasma NDEs without making any comparisons between PD and controls. They found 231 NDEs proteins shared with reference databases, and another 20 were annotated as highly expressed in the brain in the Human Protein Atlas.^1^

In short, α-synuclein in CNS-derived EVs has been the most extensively explored as a biomarker for PD due to its crucial role in disease pathology. However, there is currently a lack of research exploring biomarkers in ADEs for PD diagnosis.

### 3.2. Psychiatric disorders

In terms of studies of EVs in psychiatric disorders, only publications on SCZ, BD, and depression have been retrieved. The etiology of psychiatric disorders remains unclear, which has hindered the development of precise diagnostic biomarkers. Current research primarily focuses on cognitive function, neuroinflammatory dysfunction, and certain pathological abnormalities shared with neurodegenerative disorders.

SCZ is a major neuropsychiatric disorder affecting ~1% of the population worldwide (Kahn et al., 2015). Only four studies have included NDEs/ADEs in SCZ clinical diagnosis. These studies examined insulin resistance-related proteins (Kapogiannis et al., 2019a), complement and mitochondrial electron transport proteins (Goetzl et al., 2020, 2021a), and cognitive dysfunction-related proteins (Lee et al., 2020) in NDEs/ADEs of SCZ. The findings from these studies indicated that SCZ patients had lower NDEs levels of pS312-IRS-1, mitochondrial electron transport system proteins, and mitochondrial proteins compared to HC. SCZ patients exhibited higher ADEs levels of complement mediators and Aβ42. However, there are currently no genome-wide studies focusing on SCZ.

From the literature we collected, there have been no clinical diagnosis-related studies on BD yet. Only one study investigated drugs related to insulin signaling for the clinical treatment of BD using CNS-derived EVs as a tool (Mansur et al., 2021). This study found that plasma NDEs levels of p-ERK1/2, p-JNK, and p-p38-MAPK were significantly higher in BD patients treated with infliximab for 6 or 12 weeks but not for 2 weeks compared to placebo-treated patients. In addition, they observed a significant association between p-S312-IRS-1 and hippocampal volume.

To date, five studies have investigated NDEs/ADEs in depression. Major depressive disorder (MDD) has a prevalence of 4.7% worldwide (GBD 2016 DALYs and HALE Collaborators, 2017). Out of these five studies, four explored changes in cargos within NDEs. Two studies explored changes in protein molecules. They found that levels of IRS-1 were increased (Nasca et al., 2021), while levels of mitochondrial proteins mitofusin 2 (MFN2) and cyclophilin D (CYPD) (Goetzl et al., 2021b) were decreased in MDD patients compared to HC.

Another two studies investigated changes in miRNAs within NDEs but did not directly compare differences between MDD and HC. One study targeted eight previously reported miRNAs associated with depression and identified stable detection of let-7a-5p, miR-34a-5p, miR-132-3p, miR-182-5p, miR-212-3p, and miR-1202 in NDEs (Mizohata et al., 2021). The other study was carried out on 75 miRNAs by microarray and found that changes in miR-21-5p, miR-30d-5p, and miR-486-5p were associated with antidepressant drug response (Saeedi et al., 2021).

## 4. Evaluation of current methods for isolating NDEs, ADEs, and ODEs

Despite the extensive biomarker discovery research on NDEs, ADEs, and ODEs, the isolation methods for these EVs have not been thoroughly evaluated. All current methods are based on antibodies, making the specificity of both the antibodies and the antigens crucial. The methods currently used to isolate CNS-derived EVs are summarized in Table 2.

**Table 2:** Summary of methods used to isolate the CNS-derived EVs currently.

Type of EVs	Marker antibody target	Target expression in known brain cell type	Target expression in tissue	EV isolation methods	No. of paper
NDEs	L1CAM	Neuron	Brain, intestine, monocyte, testis	Precipitation + immunoaffinity	44
				Immunoaffinity	4
				Centrifugation + immunoaffinity	3
				Size exclusion chromatography + immunoaffinity	1
	NCAM	Neuron, oligodendrocyte	Brain, heart muscle	Precipitation + immunoaffinity	3
	SNAP25	Neuron	Brain, retina	Immunoaffinity	1
ADEs	GLAST	Astrocyte	Brain, heart, ovary, placenta	Precipitation + immunoaffinity	8
				Immunoaffinity	1
	AQP4/GFAP	Astrocyte	Brain, lung/brain, kidney	Centrifugation + flow cytometry	1
ODEs	OMG	Oligodendrocyte, Astrocyte	Brain, choroid plexus	Immunoaffinity	1
	CNP	Oligodendrocyte	Brain, retina	Immunoaffinity	1
	MOG	Oligodendrocyte	Brain, spinal cord	Precipitation + immunoaffinity	1

L1CAM is the most commonly used antigen for capturing NDEs. It is a member of the cell adhesion molecules mainly expressed in CNS and has been identified as a marker on the surface of EVs specifically derived from neurons (Fauré et al., 2006; Shi et al., 2014). According to the sorted brain cell RNAseq database,^2^ L1CAM is specifically expressed in neurons in the brain (Zhang et al., 2016). However, L1CAM is not only expressed in the brain but also the intestine, monocytes, testis,^3^ and some cancer cells (Ganesh et al., 2020). Additionally, a recent study found that L1CAM has soluble fragments in the blood that are not associated with EVs (Norman et al., 2021). These observations have raised concerns about using L1CAM as a specific marker for NDEs.

Besides L1CAM, Neural Cell Adhesion Molecule 1 (NCAM1) (Fiandaca et al., 2015; Goetzl et al., 2015a) and SNAP25 (Ohmichi et al., 2019) have also been used for the capture of NDEs. However, NCAM1 is not neuron-specific and is also highly expressed in oligodendrocytes (Sharma et al., 2015; Zhang et al., 2016) and heart muscle (Ackermann et al., 2017). On the other hand, SNAP25 exhibits good cell type and tissue specificity (Hwang and Lee, 2003), but its abundance in EVs has not been fully validated. To capture SNAP25-binding EVs in serum-free conditions, anti-SNAP25 antibodies are used, followed by the quantification of SNAP25+ EVs using anti-CD81 since CD81 is a marker of EVs.

Most methods for capturing ADEs utilize anti-GLAST antibodies, as GLAST is considered to be predominantly expressed in astrocytes. However, GLAST is also moderately expressed in the heart (Xie et al., 2022), ovary, and placenta (see text footnote 3). Aquaporin 4 (AQP4) and Glial Fibrillary Acidic Protein (GFAP), two classical astrocyte markers, have been tested for capturing ADEs by flow cytometry. Although AQP4 and GFAP exhibit good cell type specificity in the brain, they are also expressed in other organs, such as the lungs (Jaskiewicz et al., 2022) and kidneys (Buniatian et al., 1998).

The isolation of ODEs has been reported in three studies, but the justification for the choice of antibodies used in these studies is unclear. Each study employed distinct antibodies to capture ODEs, including anti-oligodendrocyte-myelin glycoprotein (OMG) (Ohmichi et al., 2019), anti-myelin oligodendrocyte glycoprotein (MOG) (Dutta et al., 2021), and CNP (Yu et al., 2020). CNP is a commonly used marker for mature oligodendrocytes and is mainly expressed in the CNS (Sprinkle, 1989; Verrier et al., 2013). CNP and MOG are both specifically highly expressed in oligodendrocytes, while OMG has been found to be expressed in astrocytes as well (Zhang et al., 2016).

The specificity of these antibodies needs to be further evaluated, as the concern regarding their specificity is not addressed in most of these papers. It is necessary to trace the main source of these EVs, such as the genetic source tracking research conducted for human urinary exosomes (Zhu et al., 2021). A comprehensive comparison of blood CNS-derived EVs with brain EVs or CSF EVs would be valuable. Also, the cargos of EVs can provide insights into their origin. The specificity of L1CAM+ EVs was evaluated (Goetzl et al., 2016b; Mustapic et al., 2017), and they were found to be highly enriched for neural proteins such as neurofilament light chain (NF-Lch) and neuron-specific enolase (NSE). The molecular signatures of blood total EVs, L1CAM+ EVs (NDEs), and epithelial cell adhesion molecule (EpCAM) + EVs (tumor-derived exosomes, another subtype in blood EVs) were compared by protein antibody array and showed distinct protein profiles. Another study reported that several biomarkers, total tau and p-T181-tau, showed a positive correlation in L1CAM+ EVs and CSF, while Aβ42 showed a negative correlation (Jia et al., 2019). Although NCAM+ EVs have not been found to contain these neural-specific cargos, Fiandaca et al. (2015) found that total tau, p-S396-tau, p-T181-tau, and Aβ42 have a similar concentration in NCAM+ EVs as they do in L1CAM+ EVs. This suggests that L1CAM+ EVs and NCAM+ EVs may have the same origin. GLAST+ EVs were found to contain astrocyte-specific proteins GFAP and GluSyn. Taken together, although L1CAM and GLAST are not specific to the brain, L1CAM+ EVs and GLAST+ EVs are enriched in neuron- and astrocyte-specific proteins, respectively. Other antigen-captured EVs have not been evaluated for their cell types or brain specificity.

The most commonly employed method for capturing CNS-derived EVs is precipitation coupled with antibody-based immunoaffinity capture, although some researchers have utilized direct antibody immunoaffinity isolation to capture L1CAM+ EVs and CNP+ EVs. While the direct immunoaffinity method is straightforward, there is a concern that soluble fragments may compromise the purity of EVs. Another approach involves centrifugation (not ultra-centrifugation) to eliminate cell debris and then couple it with antibody immunoaffinity. This procedure also poses the same concern regarding soluble fragments. The precipitation step only captures EVs and polymeric substances (Zarovni et al., 2015), which can help to remove the soluble antigen. In sum, further development is necessary to determine the source of CNS-derived EVs and assess the similarity in content between CNS-derived EVs in blood and EVs in CSF.

The methods used in these studies to identify and characterize EVs included transmission electron microscopy (TEM), nanoparticle tracking analysis (NTA), bicinchoninic acid (BCA) protein quantification, Western Blotting, tunable resistive pulse-sensing (TRPS) analysis, and other. To ensure the purity and integrity of isolated EVs, the International Society for Extracellular Vesicles (ISEV) has issued guidelines for identifying exosomes (Théry et al., 2018). However, many of the studies described in this review did not adhere to these guidelines adequately, making it challenging to determine whether there is an impurity in the isolated EVs that could interfere with the present findings. Therefore, in future studies, researchers should focus on selecting antibodies targeting specific proteins, evaluating the source, and improving the purity of the isolated CNS-derived EVs. This may help establish the association between EVs and the CNS and enhance their clinical diagnostic value.

## 5. Conclusion and future perspectives

In this review, we summarized the research progress in the diagnosis of six major brain disorders using the study of NDEs, ADEs, and ODEs. Most of these studies were conducted on neurodegenerative disorders, and many of these CNS-derived EVs’ cargos were found to be significantly different between cases and controls. It is worth mentioning that some of the detected molecules in NDEs, including Aβ42, total tau, p-T181-tau, p-S396-tau, NRGN, synaptotagmin, GAP43, SNAP25, cathepsin D, REST and α-synuclein, have been reproducible across different studies, which is of great significance for the clinical diagnosis of diseases. In addition, these peripherally detected changes in EVs represent biological alterations occurring in the brain in a minimally invasive manner, making CNS-derived EVs ideal diagnostic and therapeutic tools. These findings may drive the development of accurate clinical diagnosis for brain disorders. However, the features of CNS-derived EVs and their isolation methods need to be improved, particularly their specificity.

The specificity of many markers of the CNS-derived EVs has been questioned, and it remains to be carefully investigated whether these EVs and their cargos accurately reflect brain conditions. It is necessary to measure and compare the expression levels of the selective specific antigens in EVs to those from the relevant tissues and cell types, either in animal models or in postmortem or surgery tissues. The development and application of EVs tracer techniques also help to evaluate the specific EVs trajectories precisely. The detailed benchmark for CNS-derived EVs may facilitate clinical application and explain current inconsistent results to a certain extent.

Except that, most current studies of CNS-derived EVs only detected specific biomolecules of interest, and these results have yet to undergo validation by other research teams. A limited number of papers have used genome-wide approaches to investigate the expression pattern of CNS-derived EVs (Serpente et al., 2020; Zou et al., 2020; Anastasi et al., 2021; Arioz et al., 2021; Saeedi et al., 2021; Zhong et al., 2021). These studies have employed omics screening to predict disease status and have revealed new potential protein or miRNA biomarkers. For diseases that lack clear pathological features, such as psychiatric disorders, exploration at the omics level is necessary to identify molecular changes that can aid in clinical diagnosis or molecular typing. Therefore, it is essential to conduct additional omics-related research to discover alterations at the molecular level in CNS-derived EVs for biomarker identification. Machine learning algorithms with large omics data may help uncover better biomarkers than those that have been previously tested. While the majority of current studies concentrate on proteins, it is worth mentioning that RNA levels are also important for disease diagnosis and cannot be ignored in future studies.

In addition, compared with the disease diagnosis, early detection may be clinically more important. In the current literature collected, only a few studies related to AD have considered the pattern of biomarkers in pre-clinical samples. Therefore, researchers should carry out more pre-clinical studies on related diseases or explore the biomarkers identified in the disease’s early stages.

To summarize, it is crucial to validate the findings of specific biomolecules in CNS-derived EVs through collaboration with other research teams. Employing genome-wide approaches, considering RNA levels, conducting pre-clinical studies, and utilizing machine learning algorithms can significantly enhance the discovery of robust and reliable biomarkers for disease diagnosis and classification in CNS-derived EVs.

## Author contributions

XW, KL, and HY did the literature collection and research results collation. XW and KL conceived the manuscript. XW did the manuscript writing. KL and CL helped with manuscript writing and finalized the manuscript. All authors contributed to the article and approved the submitted version.

## Funding

This work was supported by the Startup Foundation for Introducing Talent of Central South University (202044006).

## Conflict of interest

The authors declare that the research was conducted in the absence of any commercial or financial relationships that could be construed as a potential conflict of interest.

## Publisher’s note

All claims expressed in this article are solely those of the authors and do not necessarily represent those of their affiliated organizations, or those of the publisher, the editors and the reviewers. Any product that may be evaluated in this article, or claim that may be made by its manufacturer, is not guaranteed or endorsed by the publisher.

## Footnotes

^1^ https://www.proteinatlas.org/

^2^ https://www.brainrnaseq.org/

^3^ https://www.genecards.org/

## References

[ref1] Abner E. L. Jicha G. A. Shaw L. M. Trojanowski J. Q. Goetzl E. J. (2016). Plasma neuronal exosomal levels of Alzheimer’s disease biomarkers in normal aging. Ann. Clin. Transl. Neurol. 3, 399–403. doi: 10.1002/acn3.309, PMID: 27231710 PMC4863753

[ref2] Ackermann M. A. Petrosino J. M. Manring H. R. Wright P. Shettigar V. Kilic A.. (2017). TGF-β1 affects cell-cell adhesion in the heart in an NCAM1-dependent mechanism. J. Mol. Cell. Cardiol. 112, 49–57. doi: 10.1016/j.yjmcc.2017.08.015, PMID: 28870505 PMC5647243

[ref3] Agliardi C. Guerini F. R. Zanzottera M. Bianchi A. Nemni R. Clerici M. (2019). SNAP-25 in serum is carried by exosomes of neuronal origin and is a potential biomarker of Alzheimer’s disease. Mol. Neurobiol. 56, 5792–5798. doi: 10.1007/s12035-019-1501-x, PMID: 30680692

[ref4] Agliardi C. Meloni M. Guerini F. R. Zanzottera M. Bolognesi E. Baglio F.. (2021). Oligomeric α-Syn and SNARE complex proteins in peripheral extracellular vesicles of neural origin are biomarkers for Parkinson’s disease. Neurobiol. Dis. 148:105185. doi: 10.1016/j.nbd.2020.105185 33217562

[ref5] Anastasi F. Masciandaro S. M. Carratore R. D. Dell’Anno M. T. Signore G. Falleni A.. (2021). Proteomics profiling of neuron-derived small extracellular vesicles from human plasma: enabling single-subject analysis. Int. J. Mol. Sci. 22:2951. doi: 10.3390/ijms22062951, PMID: 33799461 PMC7999506

[ref6] Arioz B. I. Tufekci K. U. Olcum M. Durur D. Y. Akarlar B. A. Ozlu N.. (2021). Proteome profiling of neuron-derived exosomes in Alzheimer’s disease reveals hemoglobin as a potential biomarker. Neurosci. Lett. 755:135914. doi: 10.1016/j.neulet.2021.135914, PMID: 33901610

[ref7] Athauda D. Gulyani S. Karnati H. K. Li Y. Tweedie D. Mustapic M.. (2019). Utility of neuronal-derived exosomes to examine molecular mechanisms that affect motor function in patients with Parkinson disease: a secondary analysis of the Exenatide-PD trial. JAMA Neurol. 76, 420–429. doi: 10.1001/jamaneurol.2018.4304, PMID: 30640362 PMC6459135

[ref8] Banks W. A. Sharma P. Bullock K. M. Hansen K. M. Ludwig N. Whiteside T. L. (2020). Transport of extracellular vesicles across the blood-brain barrier: brain pharmacokinetics and effects of inflammation. Int. J. Mol. Sci. 21:E4407. doi: 10.3390/ijms21124407, PMID: 32575812 PMC7352415

[ref9] Braak H. Del Tredici K. (2017). Neuropathological staging of brain pathology in sporadic Parkinson’s disease: separating the wheat from the chaff. J. Parkinsons Dis. 7, S71–S85. doi: 10.3233/JPD-179001, PMID: 28282810 PMC5345633

[ref10] Buniatian G. Traub P. Albinus M. Beckers G. Buchmann A. Gebhardt R.. (1998). The immunoreactivity of glial fibrillary acidic protein in mesangial cells and podocytes of the glomeruli of rat kidney in vivo and in culture. Biol. Cell. 90, 53–61. doi: 10.1016/s0248-4900(98)80232-3, PMID: 9691426

[ref11] Burré J. Sharma M. Tsetsenis T. Buchman V. Etherton M. R. Südhof T. C. (2010). Alpha-synuclein promotes SNARE-complex assembly in vivo and in vitro. Science 329, 1663–1667. doi: 10.1126/science.1195227, PMID: 20798282 PMC3235365

[ref12] Casaletto K. B. Elahi F. M. Bettcher B. M. Neuhaus J. Bendlin B. B. Asthana S.. (2017). Neurogranin, a synaptic protein, is associated with memory independent of Alzheimer biomarkers. Neurology 89, 1782–1788. doi: 10.1212/WNL.0000000000004569, PMID: 28939668 PMC5664306

[ref13] Cha D. J. Mengel D. Mustapic M. Liu W. Selkoe D. J. Kapogiannis D.. (2019). miR-212 and miR-132 are downregulated in Neurally derived plasma exosomes of Alzheimer’s patients. Front. Neurosci. 13:1208. doi: 10.3389/fnins.2019.01208, PMID: 31849573 PMC6902042

[ref14] Chi H. Yao R. Sun C. Leng B. Shen T. Wang T.. (2022). Blood Neuroexosomal mitochondrial proteins predict Alzheimer disease in diabetes. Diabetes 71, 1313–1323. doi: 10.2337/db21-0969 35287177

[ref15] Chou S.-Y. Chan L. Chung C.-C. Chiu J.-Y. Hsieh Y.-C. Hong C.-T. (2020). Altered insulin receptor substrate 1 phosphorylation in blood neuron-derived extracellular vesicles from patients with Parkinson’s disease. Front. Cell Dev. Biol. 8:564641. doi: 10.3389/fcell.2020.564641, PMID: 33344443 PMC7744811

[ref16] Du Y. Tan W.-L. Chen L. Yang Z.-M. Li X.-S. Xue X.. (2021). Exosome transplantation from patients with schizophrenia causes schizophrenia-relevant behaviors in mice: an integrative multi-omics data analysis. Schizophr. Bull. 47, 1288–1299. doi: 10.1093/schbul/sbab039, PMID: 33837780 PMC8379541

[ref17] Dubois B. Feldman H. H. Jacova C. Hampel H. Molinuevo J. L. Blennow K.. (2014). Advancing research diagnostic criteria for Alzheimer’s disease: the IWG-2 criteria. Lancet Neurol. 13, 614–629. doi: 10.1016/S1474-4422(14)70090-0, PMID: 24849862

[ref18] Dubois B. Villain N. Frisoni G. B. Rabinovici G. D. Sabbagh M. Cappa S.. (2021). Clinical diagnosis of Alzheimer’s disease: recommendations of the international working group. Lancet Neurol. 20, 484–496. doi: 10.1016/S1474-4422(21)00066-1, PMID: 33933186 PMC8339877

[ref19] Dugger B. N. Dickson D. W. (2017). Pathology of neurodegenerative diseases. Cold Spring Harb. Perspect. Biol. 9:a028035. doi: 10.1101/cshperspect.a028035, PMID: 28062563 PMC5495060

[ref20] Dutta S. Hornung S. Kruayatidee A. Maina K. N. Del Rosario I. Paul K. C.. (2021). α-synuclein in blood exosomes immunoprecipitated using neuronal and oligodendroglial markers distinguishes Parkinson’s disease from multiple system atrophy. Acta Neuropathol. 142, 495–511. doi: 10.1007/s00401-021-02324-0, PMID: 33991233 PMC8357708

[ref21] Emmanouilidou E. Melachroinou K. Roumeliotis T. Garbis S. D. Ntzouni M. Margaritis L. H.. (2010). Cell-produced alpha-synuclein is secreted in a calcium-dependent manner by exosomes and impacts neuronal survival. J. Neurosci. 30, 6838–6851. doi: 10.1523/JNEUROSCI.5699-09.2010, PMID: 20484626 PMC3842464

[ref22] Fauré J. Lachenal G. Court M. Hirrlinger J. Chatellard-Causse C. Blot B.. (2006). Exosomes are released by cultured cortical neurones. Mol. Cell. Neurosci. 31, 642–648. doi: 10.1016/j.mcn.2005.12.003 16446100

[ref23] Fevrier B. Vilette D. Archer F. Loew D. Faigle W. Vidal M.. (2004). Cells release prions in association with exosomes. Proc. Natl. Acad. Sci. U. S. A. 101, 9683–9688. doi: 10.1073/pnas.0308413101, PMID: 15210972 PMC470735

[ref24] Fiandaca M. S. Kapogiannis D. Mapstone M. Boxer A. Eitan E. Schwartz J. B.. (2015). Identification of preclinical Alzheimer’s disease by a profile of pathogenic proteins in neurally derived blood exosomes: a case-control study. Alzheimers Dement. J. Alzheimers Assoc. 11, 600–607.e1. doi: 10.1016/j.jalz.2014.06.008, PMID: 25130657 PMC4329112

[ref25] Fu Y. Jiang C. Tofaris G. K. Davis J. J. (2020). Facile impedimetric analysis of neuronal exosome markers in Parkinson’s disease diagnostics. Anal. Chem. 92, 13647–13651. doi: 10.1021/acs.analchem.0c03092, PMID: 32945162 PMC7584333

[ref26] Ganesh K. Basnet H. Kaygusuz Y. Laughney A. M. He L. Sharma R.. (2020). L1CAM defines the regenerative origin of metastasis-initiating cells in colorectal cancer. Nat. Cancer 1, 28–45. doi: 10.1038/s43018-019-0006-x, PMID: 32656539 PMC7351134

[ref27] GBD 2016 DALYs and HALE Collaborators (2017). Global, regional, and national disability-adjusted life-years (DALYs) for 333 diseases and injuries and healthy life expectancy (HALE) for 195 countries and territories, 1990-2016: a systematic analysis for the global burden of disease study 2016. Lancet Lond. Engl. 390, 1260–1344. doi: 10.1016/S0140-6736(17)32130-X, PMID: 28919118 PMC5605707

[ref28] Goetzl E. J. Abner E. L. Jicha G. A. Kapogiannis D. Schwartz J. B. (2018a). Declining levels of functionally specialized synaptic proteins in plasma neuronal exosomes with progression of Alzheimer’s disease. FASEB. J. Off. Publ. Fed. Am. Soc. Exp. Biol. 32, 888–893. doi: 10.1096/fj.201700731R, PMID: 29025866 PMC5888398

[ref29] Goetzl E. J. Boxer A. Schwartz J. B. Abner E. L. Petersen R. C. Miller B. L.. (2015b). Low neural exosomal levels of cellular survival factors in Alzheimer’s disease. Ann. Clin. Transl. Neurol. 2, 769–773. doi: 10.1002/acn3.211, PMID: 26273689 PMC4531059

[ref30] Goetzl E. J. Boxer A. Schwartz J. B. Abner E. L. Petersen R. C. Miller B. L.. (2015a). Altered lysosomal proteins in neural-derived plasma exosomes in preclinical Alzheimer disease. Neurology 85, 40–47. doi: 10.1212/WNL.0000000000001702, PMID: 26062630 PMC4501943

[ref31] Goetzl E. J. Kapogiannis D. Schwartz J. B. Lobach I. V. Goetzl L. Abner E. L.. (2016a). Decreased synaptic proteins in neuronal exosomes of frontotemporal dementia and Alzheimer’s disease. FASEB J. Off. Publ. Fed. Am. Soc. Exp. Biol. 30, 4141–4148. doi: 10.1096/fj.201600816R, PMID: 27601437 PMC5102122

[ref32] Goetzl E. J. Mustapic M. Kapogiannis D. Eitan E. Lobach I. V. Goetzl L.. (2016b). Cargo proteins of plasma astrocyte-derived exosomes in Alzheimer’s disease. FASEB. J. Off. Publ. Fed. Am. Soc. Exp. Biol. 30, 3853–3859. doi: 10.1096/fj.201600756R PMC5067254 27511944

[ref33] Goetzl E. J. Nogueras-Ortiz C. Mustapic M. Mullins R. J. Abner E. L. Schwartz J. B.. (2019). Deficient neurotrophic factors of CSPG4-type neural cell exosomes in Alzheimer disease. FASEB J. Off. Publ. Fed. Am. Soc. Exp. Biol. 33, 231–238. doi: 10.1096/fj.201801001, PMID: 29924942 PMC6355088

[ref34] Goetzl E. J. Schwartz J. B. Abner E. L. Jicha G. A. Kapogiannis D. (2018b). High complement levels in astrocyte-derived exosomes of Alzheimer disease. Ann. Neurol. 83, 544–552. doi: 10.1002/ana.25172, PMID: 29406582 PMC5867263

[ref35] Goetzl E. J. Srihari V. H. Guloksuz S. Ferrara M. Tek C. Heninger G. R. (2020). Decreased mitochondrial electron transport proteins and increased complement mediators in plasma neural-derived exosomes of early psychosis. Transl. Psychiatry 10:361. doi: 10.1038/s41398-020-01046-3, PMID: 33106473 PMC7588411

[ref36] Goetzl E. J. Srihari V. H. Guloksuz S. Ferrara M. Tek C. Heninger G. R. (2021a). Neural cell-derived plasma exosome protein abnormalities implicate mitochondrial impairment in first episodes of psychosis. FASEB J. Off. Publ. Fed. Am. Soc Exp. Biol. 35:e21339. doi: 10.1096/fj.202002519R 33454965

[ref37] Goetzl E. J. Wolkowitz O. M. Srihari V. H. Reus V. I. Goetzl L. Kapogiannis D.. (2021b). Abnormal levels of mitochondrial proteins in plasma neuronal extracellular vesicles in major depressive disorder. Mol. Psychiatry 26, 7355–7362. doi: 10.1038/s41380-021-01268-x, PMID: 34471251 PMC8872999

[ref38] Gu D. Liu F. Meng M. Zhang L. Gordon M. L. Wang Y.. (2020). Elevated matrix metalloproteinase-9 levels in neuronal extracellular vesicles in Alzheimer’s disease. Ann. Clin. Transl. Neurol. 7, 1681–1691. doi: 10.1002/acn3.51155, PMID: 32790155 PMC7480907

[ref39] Guix F. X. Corbett G. T. Cha D. J. Mustapic M. Liu W. Mengel D.. (2018). Detection of aggregation-competent tau in neuron-derived extracellular vesicles. Int. J. Mol. Sci. 19:E663. doi: 10.3390/ijms19030663, PMID: 29495441 PMC5877524

[ref40] Harding C. Stahl P. (1983). Transferrin recycling in reticulocytes: pH and iron are important determinants of ligand binding and processing. Biochem. Biophys. Res. Commun. 113, 650–658. doi: 10.1016/0006-291x(83)91776-x, PMID: 6870878

[ref41] Hwang S. B. Lee J. (2003). Neuron cell type-specific SNAP-25 expression driven by multiple regulatory elements in the nematode Caenorhabditis elegans. J. Mol. Biol. 333, 237–247. doi: 10.1016/j.jmb.2003.08.055 14529613

[ref42] Jack C. R. Bennett D. A. Blennow K. Carrillo M. C. Dunn B. Haeberlein S. B.. (2018). NIA-AA research framework: toward a biological definition of Alzheimer’s disease. Alzheimers Dement. J. Alzheimers Assoc. 14, 535–562. doi: 10.1016/j.jalz.2018.02.018, PMID: 29653606 PMC5958625

[ref43] Jaskiewicz L. Hejne K. Szostak B. Osowiecka K. Skowronski M. T. Lepiarczyk E.. (2022). Expression profiles of AQP3 and AQP4 in lung adenocarcinoma samples generated via Bronchoscopic biopsies. J. Clin. Med. 11:5954. doi: 10.3390/jcm11195954, PMID: 36233821 PMC9573329

[ref44] Jia L. Qiu Q. Zhang H. Chu L. Du Y. Zhang J.. (2019). Concordance between the assessment of Aβ42, T-tau, and P-T181-tau in peripheral blood neuronal-derived exosomes and cerebrospinal fluid. Alzheimers Dement. J. Alzheimers Assoc. 15, 1071–1080. doi: 10.1016/j.jalz.2019.05.002, PMID: 31422798

[ref45] Jia L. Zhu M. Kong C. Pang Y. Zhang H. Qiu Q.. (2021). Blood neuro-exosomal synaptic proteins predict Alzheimer’s disease at the asymptomatic stage. Alzheimers Dement. J. Alzheimers Assoc. 17, 49–60. doi: 10.1002/alz.12166, PMID: 32776690 PMC7984076

[ref46] Jiang C. Hopfner F. Berg D. Hu M. T. Pilotto A. Borroni B.. (2021). Validation of α-synuclein in L1CAM-Immunocaptured exosomes as a biomarker for the stratification of parkinsonian syndromes. Mov. Disord. Off. J. Mov. Disord. Soc. 36, 2663–2669. doi: 10.1002/mds.28591, PMID: 33826157 PMC8663480

[ref47] Jiang C. Hopfner F. Katsikoudi A. Hein R. Catli C. Evetts S.. (2020). Serum neuronal exosomes predict and differentiate Parkinson’s disease from atypical parkinsonism. J. Neurol. Neurosurg. Psychiatry 91, 720–729. doi: 10.1136/jnnp-2019-322588, PMID: 32273329 PMC7361010

[ref48] Kahn R. S. Sommer I. E. Murray R. M. Meyer-Lindenberg A. Weinberger D. R. Cannon T. D.. (2015). Schizophrenia. Nat. Rev. Dis. Primer 1:15067. doi: 10.1038/nrdp.2015.67 27189524

[ref49] Kapogiannis D. Dobrowolny H. Tran J. Mustapic M. Frodl T. Meyer-Lotz G.. (2019a). Insulin-signaling abnormalities in drug-naïve first-episode schizophrenia: transduction protein analyses in extracellular vesicles of putative neuronal origin. Eur. Psychiatry J. Assoc. Eur. Psychiatr. 62, 124–129. doi: 10.1016/j.eurpsy.2019.08.012, PMID: 31590015 PMC6941668

[ref50] Kapogiannis D. Mustapic M. Shardell M. D. Berkowitz S. T. Diehl T. C. Spangler R. D.. (2019b). Association of extracellular vesicle biomarkers with Alzheimer disease in the Baltimore longitudinal study of aging. JAMA Neurol. 76, 1340–1351. doi: 10.1001/jamaneurol.2019.2462, PMID: 31305918 PMC6632160

[ref51] Krämer-Albers E.-M. Bretz N. Tenzer S. Winterstein C. Möbius W. Berger H.. (2007). Oligodendrocytes secrete exosomes containing major myelin and stress-protective proteins: trophic support for axons? Proteomics Clin. Appl. 1, 1446–1461. doi: 10.1002/prca.200700522, PMID: 21136642

[ref52] Kvartsberg H. Duits F. H. Ingelsson M. Andreasen N. Öhrfelt A. Andersson K.. (2015). Cerebrospinal fluid levels of the synaptic protein neurogranin correlates with cognitive decline in prodromal Alzheimer’s disease. Alzheimers Dement. J. Alzheimers Assoc. 11, 1180–1190. doi: 10.1016/j.jalz.2014.10.009, PMID: 25533203

[ref53] Lee E. E. Winston-Gray C. Barlow J. W. Rissman R. A. Jeste D. V. (2020). Plasma levels of neuron- and astrocyte-derived Exosomal amyloid Beta1-42, amyloid Beta1-40, and phosphorylated tau levels in schizophrenia patients and non-psychiatric comparison subjects: relationships with cognitive functioning and psychopathology. Front. Psych. 11:532624. doi: 10.3389/fpsyt.2020.532624, PMID: 33762974 PMC7982803

[ref54] Liberati A. Altman D. G. Tetzlaff J. Mulrow C. Gøtzsche P. C. Ioannidis J. P. A.. (2009). The PRISMA statement for reporting systematic reviews and meta-analyses of studies that evaluate healthcare interventions: explanation and elaboration. BMJ 339:b2700. doi: 10.1136/bmj.b2700, PMID: 19622552 PMC2714672

[ref55] Mansur R. B. Delgado-Peraza F. Subramaniapillai M. Lee Y. Iacobucci M. Nasri F.. (2021). Exploring brain insulin resistance in adults with bipolar depression using extracellular vesicles of neuronal origin. J. Psychiatr. Res. 133, 82–92. doi: 10.1016/j.jpsychires.2020.12.007, PMID: 33316649 PMC7855678

[ref56] Mathieu M. Martin-Jaular L. Lavieu G. Théry C. (2019). Specificities of secretion and uptake of exosomes and other extracellular vesicles for cell-to-cell communication. Nat. Cell Biol. 21, 9–17. doi: 10.1038/s41556-018-0250-9, PMID: 30602770

[ref57] Mizohata Y. Toda H. Koga M. Saito T. Fujita M. Kobayashi T.. (2021). Neural extracellular vesicle-derived miR-17 in blood as a potential biomarker of subthreshold depression. Hum. Cell 34, 1087–1092. doi: 10.1007/s13577-021-00553-9, PMID: 34013455

[ref58] Morales-Prieto D. M. Murrieta-Coxca J. M. Stojiljkovic M. Diezel C. Streicher P. E. Henao-Restrepo J. A.. (2022). Small extracellular vesicles from peripheral blood of aged mice pass the blood-brain barrier and induce glial cell activation. Cells 11:625. doi: 10.3390/cells11040625, PMID: 35203276 PMC8870085

[ref59] Mustapic M. Eitan E. Werner J. K. Berkowitz S. T. Lazaropoulos M. P. Tran J.. (2017). Plasma extracellular vesicles enriched for neuronal origin: a potential window into brain pathologic processes. Front. Neurosci. 11:278. doi: 10.3389/fnins.2017.00278 28588440 PMC5439289

[ref60] Nam E. Lee Y.-B. Moon C. Chang K.-A. (2020). Serum tau proteins as potential biomarkers for the assessment of Alzheimer’s disease progression. Int. J. Mol. Sci. 21:E5007. doi: 10.3390/ijms21145007, PMID: 32679907 PMC7404390

[ref61] Nasca C. Dobbin J. Bigio B. Watson K. de Angelis P. Kautz M.. (2021). Insulin receptor substrate in brain-enriched exosomes in subjects with major depression: on the path of creation of biosignatures of central insulin resistance. Mol. Psychiatry 26, 5140–5149. doi: 10.1038/s41380-020-0804-7, PMID: 32536688 PMC7787430

[ref62] Naz F. Siddique Y. H. (2020). Human brain disorders: a review. Open Biol. J. 8, 6–21. doi: 10.2174/1874196702008010006

[ref63] Niu M. Li Y. Li G. Zhou L. Luo N. Yao M.. (2020). A longitudinal study on α-synuclein in plasma neuronal exosomes as a biomarker for Parkinson’s disease development and progression. Eur. J. Neurol. 27, 967–974. doi: 10.1111/ene.14208 32150777

[ref64] Nonaka T. Masuda-Suzukake M. Arai T. Hasegawa Y. Akatsu H. Obi T.. (2013). Prion-like properties of pathological TDP-43 aggregates from diseased brains. Cell Rep. 4, 124–134. doi: 10.1016/j.celrep.2013.06.007, PMID: 23831027

[ref65] Norman M. Ter-Ovanesyan D. Trieu W. Lazarovits R. Kowal E. J. K. Lee J. H.. (2021). L1CAM is not associated with extracellular vesicles in human cerebrospinal fluid or plasma. Nat. Methods 18, 631–634. doi: 10.1038/s41592-021-01174-8, PMID: 34092791 PMC9075416

[ref66] Ohmichi T. Mitsuhashi M. Tatebe H. Kasai T. Ali El-Agnaf O. M. Tokuda T. (2019). Quantification of brain-derived extracellular vesicles in plasma as a biomarker to diagnose Parkinson’s and related diseases. Parkinsonism Relat. Disord. 61, 82–87. doi: 10.1016/j.parkreldis.2018.11.021, PMID: 30502924

[ref67] Opel N. Goltermann J. Hermesdorf M. Berger K. Baune B. T. Dannlowski U. (2020). Cross-disorder analysis of brain structural abnormalities in six major psychiatric disorders: a secondary analysis of mega- and meta-analytical findings from the ENIGMA consortium. Biol. Psychiatry 88, 678–686. doi: 10.1016/j.biopsych.2020.04.027, PMID: 32646651

[ref68] Pan B. T. Johnstone R. M. (1983). Fate of the transferrin receptor during maturation of sheep reticulocytes in vitro: selective externalization of the receptor. Cells 33, 967–978. doi: 10.1016/0092-8674(83)90040-5, PMID: 6307529

[ref69] Saeedi S. Nagy C. Ibrahim P. Théroux J.-F. Wakid M. Fiori L. M.. (2021). Neuron-derived extracellular vesicles enriched from plasma show altered size and miRNA cargo as a function of antidepressant drug response. Mol. Psychiatry 26, 7417–7424. doi: 10.1038/s41380-021-01255-2, PMID: 34385599

[ref70] Saman S. Kim W. Raya M. Visnick Y. Miro S. Saman S.. (2012). Exosome-associated tau is secreted in tauopathy models and is selectively phosphorylated in cerebrospinal fluid in early Alzheimer disease. J. Biol. Chem. 287, 3842–3849. doi: 10.1074/jbc.M111.277061, PMID: 22057275 PMC3281682

[ref71] Serpente M. Fenoglio C. D’Anca M. Arcaro M. Sorrentino F. Visconte C.. (2020). MiRNA profiling in plasma neural-derived small extracellular vesicles from patients with Alzheimer’s disease. Cells 9:E1443. doi: 10.3390/cells9061443, PMID: 32531989 PMC7349735

[ref72] Serrano-Pozo A. Frosch M. P. Masliah E. Hyman B. T. (2011). Neuropathological alterations in Alzheimer disease. Cold Spring Harb. Perspect. Med. 1:a006189. doi: 10.1101/cshperspect.a006189, PMID: 22229116 PMC3234452

[ref73] Sharma K. Schmitt S. Bergner C. G. Tyanova S. Kannaiyan N. Manrique-Hoyos N.. (2015). Cell type- and brain region-resolved mouse brain proteome. Nat. Neurosci. 18, 1819–1831. doi: 10.1038/nn.4160, PMID: 26523646 PMC7116867

[ref74] Shi M. Kovac A. Korff A. Cook T. J. Ginghina C. Bullock K. M.. (2016). CNS tau efflux via exosomes is likely increased in Parkinson’s disease but not in Alzheimer’s disease. Alzheimers Dement. J. Alzheimers Assoc. 12, 1125–1131. doi: 10.1016/j.jalz.2016.04.003 PMC5107127 27234211

[ref75] Shi M. Liu C. Cook T. J. Bullock K. M. Zhao Y. Ginghina C.. (2014). Plasma exosomal α-synuclein is likely CNS-derived and increased in Parkinson’s disease. Acta Neuropathol. 128, 639–650. doi: 10.1007/s00401-014-1314-y 24997849 PMC4201967

[ref76] Shults C. W. (2006). Lewy bodies. Proc. Natl. Acad. Sci. U. S. A. 103, 1661–1668. doi: 10.1073/pnas.0509567103, PMID: 16449387 PMC1413649

[ref77] Sprinkle T. J. (1989). 2′,3′-cyclic nucleotide 3′-phosphodiesterase, an oligodendrocyte-Schwann cell and myelin-associated enzyme of the nervous system. Crit. Rev. Neurobiol. 4, 235–301. PMID: 2537684

[ref78] Théry C. Boussac M. Véron P. Ricciardi-Castagnoli P. Raposo G. Garin J.. (2001). Proteomic analysis of dendritic cell-derived exosomes: a secreted subcellular compartment distinct from apoptotic vesicles. J. Immunol. Baltim. Md 166, 7309–7318. doi: 10.4049/jimmunol.166.12.7309, PMID: 11390481

[ref79] Théry C. Witwer K. W. Aikawa E. Alcaraz M. J. Anderson J. D. Andriantsitohaina R.. (2018). Minimal information for studies of extracellular vesicles 2018 (MISEV2018): a position statement of the International Society for Extracellular Vesicles and update of the MISEV2014 guidelines. J. Extracell. Vesicles 7:1535750. doi: 10.1080/20013078.2018.1535750, PMID: 30637094 PMC6322352

[ref80] Thome A. D. Harms A. S. Volpicelli-Daley L. A. Standaert D. G. (2016). microRNA-155 regulates alpha-synuclein-induced inflammatory responses in models of Parkinson disease. J. Neurosci. 36, 2383–2390. doi: 10.1523/JNEUROSCI.3900-15.2016, PMID: 26911687 PMC4764660

[ref81] Verrier J. D. Jackson T. C. Gillespie D. G. Janesko-Feldman K. Bansal R. Goebbels S.. (2013). Role of CNPase in the oligodendrocytic extracellular 2’,3’-cAMP-adenosine pathway. Glia 61, 1595–1606. doi: 10.1002/glia.22523 23922219 PMC3998092

[ref82] Wei Z.-X. Xie G.-J. Mao X. Zou X.-P. Liao Y.-J. Liu Q.-S.. (2020). Exosomes from patients with major depression cause depressive-like behaviors in mice with involvement of miR-139-5p-regulated neurogenesis. Neuropsychopharmacol. Off. Publ. Am. Coll. Neuropsychopharmacol. 45, 1050–1058. doi: 10.1038/s41386-020-0622-2, PMID: 31986519 PMC7162931

[ref83] Winston C. N. Goetzl E. J. Akers J. C. Carter B. S. Rockenstein E. M. Galasko D.. (2016). Prediction of conversion from mild cognitive impairment to dementia with neuronally derived blood exosome protein profile. Alzheimers Dement. Amst. Neth. 3, 63–72. doi: 10.1016/j.dadm.2016.04.001, PMID: 27408937 PMC4925777

[ref84] Winston C. N. Goetzl E. J. Baker L. D. Vitiello M. V. Rissman R. A. (2018). Growth hormone-releasing hormone modulation of neuronal exosome biomarkers in mild cognitive impairment. J. Alzheimers Dis. 66, 971–981. doi: 10.3233/JAD-180302, PMID: 30372675 PMC6487872

[ref85] Winston C. N. Goetzl E. J. Schwartz J. B. Elahi F. M. Rissman R. A. (2019). Complement protein levels in plasma astrocyte-derived exosomes are abnormal in conversion from mild cognitive impairment to Alzheimer’s disease dementia. Alzheimers Dement. Amst. Neth. 11, 61–66. doi: 10.1016/j.dadm.2018.11.002 PMC6477776 31032394

[ref86] Xie D. Xiong K. Su X. Wang G. Zou Q. Wang L.. (2022). Glutamate drives “local Ca2+ release” in cardiac pacemaker cells. Cell Res. 32, 843–854. doi: 10.1038/s41422-022-00693-z, PMID: 35840807 PMC9437105

[ref87] Yu Z. Shi M. Stewart T. Fernagut P.-O. Huang Y. Tian C.. (2020). Reduced oligodendrocyte exosome secretion in multiple system atrophy involves SNARE dysfunction. Brain J. Neurol. 143, 1780–1797. doi: 10.1093/brain/awaa110 PMC7296853 32428221

[ref88] Yuyama K. Yamamoto N. Yanagisawa K. (2008). Accelerated release of exosome-associated GM1 ganglioside (GM1) by endocytic pathway abnormality: another putative pathway for GM1-induced amyloid fibril formation. J. Neurochem. 105, 217–224. doi: 10.1111/j.1471-4159.2007.05128.x, PMID: 18021298

[ref89] Zarovni N. Corrado A. Guazzi P. Zocco D. Lari E. Radano G.. (2015). Integrated isolation and quantitative analysis of exosome shuttled proteins and nucleic acids using immunocapture approaches. Methods San Diego Calif 87, 46–58. doi: 10.1016/j.ymeth.2015.05.028, PMID: 26044649

[ref90] Zhang Y. Sloan S. A. Clarke L. E. Caneda C. Plaza C. A. Blumenthal P. D.. (2016). Purification and characterization of progenitor and mature human astrocytes reveals transcriptional and functional differences with mouse. Neuron 89, 37–53. doi: 10.1016/j.neuron.2015.11.013, PMID: 26687838 PMC4707064

[ref91] Zhao Z.-H. Chen Z.-T. Zhou R.-L. Zhang X. Ye Q.-Y. Wang Y.-Z. (2018). Increased DJ-1 and α-synuclein in plasma neural-derived exosomes as potential markers for Parkinson’s disease. Front. Aging Neurosci. 10:438. doi: 10.3389/fnagi.2018.00438, PMID: 30692923 PMC6339871

[ref92] Zhao A. Li Y. Yan Y. Qiu Y. Li B. Xu W.. (2020). Increased prediction value of biomarker combinations for the conversion of mild cognitive impairment to Alzheimer’s dementia. Transl. Neurodegener. 9:30. doi: 10.1186/s40035-020-00210-5, PMID: 32741361 PMC7397685

[ref93] Zhong J. Ren X. Liu W. Wang S. Lv Y. Nie L.. (2021). Discovery of novel markers for identifying cognitive decline using neuron-derived exosomes. Front. Aging Neurosci. 13:696944. doi: 10.3389/fnagi.2021.696944, PMID: 34512304 PMC8427802

[ref94] Zhu Q. Cheng L. Deng C. Huang L. Li J. Wang Y.. (2021). The genetic source tracking of human urinary exosomes. Proc. Natl. Acad. Sci. U. S. A. 118:e2108876118. doi: 10.1073/pnas.2108876118 34663731 PMC8639375

[ref95] Zitvogel L. Regnault A. Lozier A. Wolfers J. Flament C. Tenza D.. (1998). Eradication of established murine tumors using a novel cell-free vaccine: dendritic cell-derived exosomes. Nat. Med. 4, 594–600. doi: 10.1038/nm0598-594, PMID: 9585234

[ref96] Zou J. Guo Y. Wei L. Yu F. Yu B. Xu A. (2020). Long noncoding RNA POU3F3 and α-synuclein in plasma L1CAM exosomes combined with β-glucocerebrosidase activity: potential predictors of Parkinson’s disease. Neurother. J. Am. Soc. Exp. Neurother. 17, 1104–1119. doi: 10.1007/s13311-020-00842-5 PMC7609611 32236821

[ref97] Zou Y. Mu D. Ma X. Wang D. Zhong J. Gao J.. (2022). Review on the roles of specific cell-derived exosomes in Alzheimer’s disease. Front. Neurosci. 16:936760. doi: 10.3389/fnins.2022.936760, PMID: 35968378 PMC9366882

